# Hemostatic profile of infants with spontaneous prematurity: can we predict intraventricular hemorrhage development?

**DOI:** 10.1186/s13052-019-0709-8

**Published:** 2019-08-28

**Authors:** Audrey Hochart, Alexandra Nuytten, Adeline Pierache, Anne Bauters, Antoine Rauch, Bénédicte Wibaut, Sophie Susen, Jenny Goudemand

**Affiliations:** 1 CHU Lille, Hematology and Transfusion, F-59000 Lille, France; 2 Univ. Lille, EA 2694 – Epidemiology and quality of care, Lille, France; 3 Department of Neonatology, Hospital Jeanne de Flandre, CHU Lille, Lille, France

**Keywords:** Prematurity, Haemostasis, Intraventricular hemorrhage, Thrombocytopenia

## Abstract

**Background:**

Defining hemostatic profile for preterm infants is a challenge when severe bleedings are frequent.

**Methods:**

The aim was to define the hemostatic profile at birth of infants with spontaneous prematurity and to evaluate whether characteristic profiles can predict the development of intraventricular hemorrhage (IVH) in prematures.

**Results:**

We included 122 newborns with a median age of 31^5/7^ gestational age (GA) [29^2/7^;34^0/7^] and median weight of 1145 g [785;1490]. Levels of fibrinogen, factor II (FII) and factor V (FV) rose with GA (*p* = 0.017,*p* = 0.009, *p* = 0.001). In the group of 23^0/7^ – 28^6/7^ GA, the 5th percentile was defined as 0.6 g/L for fibrinogen, 15 IU/dL for FII and 16 IU/dL for factor V (*n* = 30). In the group of 29^0/7^–32^6/7^ GA, the 5th percentile was defined as 1.0 g/L for fibrinogen, 24 IU/dL for FII and 41 IU/dL for factor V (*n* = 46). In the group of 33^0/7^–36^6/7^ GA, the 5th percentile was defined as 1.0 g/L for fibrinogen, 24 IU/dL for FII and 30 IU/dL for factor V (*n* = 46). Level of fibrinogen was higher in case of vaginal delivery and lower in case of IUGR. Only lower level of FV at birth was significantly associated with IVH (63.5 [46.0; 76.5] vs 74.0 [58.0; 89.0], *p* = 0.026) with an unadjusted OR per SD increase in FV of 0.57 (95%CI, 0.34 to 0.96). After adjustment for age, the association between FV level and IVH was slightly attenuated (adjusted OR, 0.70; 95%CI, 0.40 to 1.23) but remained not significant (*p* = 0.22).There was no correlation with FII and fibrinogen.

**Conclusions:**

We can define hemostastic profile of prematures and corroborate references ranges for studied parameters. Further large studies are still called for, to correlate the grade of hemorrhage and the factor V level at birth.

## Introduction

The coagulation system of the newborn differs from that of adults, and among full-term newborn, premature newborn and fetus, the hemostatic balance is different. This is the developmental hemostasis concept [1–5].

The premature hemostatic balance is an evolving process depending on fetal hepatic maturation (increasing with gestational age) and the maturation inherent to birth (especially the effect of labour on the coagulation system) [6]. Indeed, coagulation factors do not cross the placenta and their synthesis is independent from maternal coagulation proteins synthesis.

Most data on coagulation factors in the preterm neonate’s population have been obtained from fetuses at the occasion of prenatal diagnosis. However, data from fetus are considering only fetal hepatic maturation whereas it was demonstrated that there is an effect of labour and birth itself on the coagulation system in the neonate [6, 7].

Thanks to advances in neonatal resuscitation, more extremely premature infants can survive and there is a crucial need for consensus in interpreting coagulation profile in preterm neonates [8]. Indeed, plasma is commonly transfused in neonatal intensive care units to prevent hemorrhages despite no evidence to support this, and progress could be made to improve plasma transfusion practice [9].

Among bleeding complications, intraventricular hemorrhage (IVH) is associated with an increased risk of mortality and long-term morbidity. Indeed, seizures, mental retardation and cerebral palsy have been reported in 45 to 86% of preterm neonates with IVH and even a low grades of IVH can adversely influence long-term neurodevelopmental outcomes in extremely preterm infants [10]. The potential role of the coagulopathy in IVH development is still under debate [11].

The aim of this study was to define the hemostatic profile (based on platelets, FII, FV and fibrinogen) of premature infants and to evaluate whether characteristic profiles can predict the development of IVH in premature infant. Secondary objectives were to correlate the fibrinogen, factor V and factor II values with antenatal corticosteroids, delivery, gestational age (GA), weight, intrauterine growth restriction (IUGR), biological data and the newborn outcome.

## Methods

### Study population

This retrospective observational cohort study was conducted in the neonatal intensive care unit (NICU) of the Lille University Hospital (France). Infants born between 23 and 36 weeks of gestation who had coagulation testing were eligible if diagnosed with spontaneous prematurity (without maternal or fetal infection) between January 2016 and December 2017. Exclusion criteria were preeclampsia or perinatal asphyxia context.

According to our department protocol, coagulation tests were performed within the first hour of life before vitamin K administration as part of routine laboratory testing. Coagulation testing was not exhaustive, but performed according to clinician appreciation. Coagulation tests included fibrinogen and factors II and V in order to cover the main coagulation pathway.

### Data collection

The following characteristics were collected in infants: gestational age, birth weight, administration of antenatal steroids, mode of delivery, birth fibrinogen, birth factor II, birth factor V, presence and grade of IVH. GA at delivery was determined by routine first trimester ultrasound examination. Cranial ultrasound was performed by a trained neonatologist within the first 24 h of life and control between day 3 and day 5. Cranial ultrasound was repeated every week the first month of life, and then once a month until term, if normal. Cranial ultrasounds were conducted more frequently in case of IVH grade 3 or 4 (frequency depending on evolution and complications).The time of the diagnosis of IVH was not recorded. The presence and grade of IVH were determined as part of clinical care of the patients, using the Papile classification [12]. Thrombocytopenia was defined as < 135 × 10^9^/L platelets in neonates < 28 weeks and < 140 × 10^9^/L platelets in neonates > 28 weeks.

Data from patients were retrospectively collected. In this context, we obtained a CNIL (Commission Nationale Informatique et Libertés) authorization (CNIL DEC18–342).

### Blood collection

Blood samples were obtained by umbilical vein catheter at routine immediately after birth. Blood was collected into citrated (0.109 M) tubes with a final 9:1 ratio of blood to anticoagulant followed by gentle mixing. Platelet poor plasma (PPP) was prepared by centrifugation of citrated blood at 3000 x g for 10 min at room temperature.

### Factors assays

Factor II, factor V and fibrinogen were measured in platelet poor plasma (PPP) using the fully automated STA R Max2 (Stago, France) coagulometer with Neoplastin R and Stago factor specific deficient plasma and STA liquid fibrinogen (STAGO, France). All testing were performed in accordance with manufacturer’s instructions.

### Statistical analysis

Categorical variables were expressed as frequency (percentage) and continuous variables as mean ± standard deviation (SD) or median [interquartile range] in case of non-Gaussian distribution. Normality of distribution was checked graphically and by using the Shapiro–Wilk test. For each GA subgroups (23 ^0/7^–28 ^6/7^ GA, 29 ^0/7^–32 ^6/7^ GA and 33 ^0/7^–36 ^6/7^ GA), we calculated the reference ranges defined as 5 to 95th percentiles for fibrinogen, factor II and factor V.

We assessed the associations of platelets, fibrinogen, factor II and factor V with binary variables (antenatal corticosteroids, delivery, IUGR, thrombocytopenia, hypophosphatemia and intraventricular hemorrhage (newborn outcome)) using Student t or Mann-Whitney U tests (regarding the normality of distributions), with 3-levels categorical variable (antenatal corticosteroid) using Kruskall-Wallis tests, and with continuous variables (GA and, weight,) IUGR, biological data and with the newborn outcome in univariate analyses. The Mann-Whitney U test was used for binary categorical variables, a Kruskall-Wallis test for 3-levels categorical variable and using Spearman’s rank coefficient correlation for continuous variables.

Finally, the association between of thrombocytopenia and intraventricular hemorrhage was studied using the Chi-square test. Given that age is a main confounder in association between factor V and intraventricular hemorrhage, we performed a multivariable logistic regression model to adjust this association for age; odds ratio (OR) of intraventricular hemorrhage per 1 SD increase in factor V was calculated as effect size.

## Results

### Population

Among 142 eligible premature newborns, 122 were included (18 were excluded in a preeclampsia context and 2 because of perinatal asphyxia).

Median age was 31^5/7^ GA [29^2/7^; 34^0/7^] and median weight was 1145 g [785; 1490]. Among premature, 42.6% (*n* = 52) did not have IUGR. Regarding the mode of delivery, 27.0% (*n* = 33) were vaginally delivered and 73.0% (*n* = 89) were caesarean delivery (Table 1).

**Table 1:** Population (*n* = 122)

Gestational age	31^5/7^ [29^2/7^; 34^0/7^]
Weight, grams^a^	1145 [785; 1490]
Intrauterine growth restriction	52 (42.6%)
Caesarean delivery	89 (73.0%)
Prenatal corticosteroids partial course	82 (67.2%)
Prenatal corticosteroids complete course	24 (19.7%)
Hypophosphatemia	42 (34.4%)
Thrombocytopenia	36 (29.5%)
Intra ventricular hemorrhage	28 (23.0%)
Red Blood Cells transfusions	44 (36.1%)
Fresh frozen plasma	13 (10.7%)
Mortality	4 (3.3%)

Values are expressed as median [interquartile range] or no. (percentage)

^a^ 3 missing values

Among mothers, 13.1% (*n* = 16) did not received prenatal corticosteroids, 67.2% (*n* = 82) were exposed to a partial course of antenatal corticosteroids and 19.7% (*n* = 24) were exposed to a complete course of antenatal corticosteroids. None of the mother received medications like anticoagulants or antiepleptics and none of the mothers presented with thrombocytopenia related with immune disorder.

Regarding biological data, 34.4% (*n* = 42) of newborns presented with hypophosphatemia and 29.5% (*n* = 36) with thrombocytopenia. Mean number of platelets was 181 × 10^9^/L (77.5).

IVH was diagnosed in 23.0% of premature (*n* = 28) and classified as grade I (*n* = 8), grade II (*n* = 15), grade III (*n* = 3) and grade IV (*n* = 2).

Among premature, 36.1% (*n* = 44) received one or more red blood cell (RBC) transfusions (maximum 9) and 10.7% (*n* = 13) received fresh frozen plasma (15 ml/kg). Four premature (3.3%) died in the NICU (Table 1).

Among the 89 newborns with available data, 47 did not develop secondary sepsis, 13 were suspected of sepsis with no bacteriological data and 29 developed sepsis with bacteriological documentation.

Among the 89 newborns with available data, 27 were medically treated by Ibuprofen to induce closure of the ductus and 4 were surgically treated to induce closure of the ductus.

### Correlations with antenatal corticosteroids, delivery, GA, weight and IUGR

Levels of fibrinogen, factor II and factor V did not differ significantly according to the use of antenatal corticosteroids (Fibrinogen *p* = 0.49, II *p* = 0.14 and V *p* = 0.050).

Level of fibrinogen was higher in case of vaginal delivery compare to caesarean (1.8 g/L [1.4; 2.4] vs 1.6 g/L [1.2; 1.8], *p* = 0.027). There was no difference regarding factors II and V.

Levels of fibrinogen, factor II and factor V rose with GA (*p* = 0.017, *p* = 0.009, *p* = 0.001, respectively) and with increasing birth weight (*p* < 0.001 *p* = 0.001 and *p* < 0.001, respectively). In the group of 23 ^0/7^–28 ^6/7^ GA, the 5th percentile was defined as 0.6 g/L for fibrinogen, 15 IU/dL for FII and 16 IU/dL for factor V (*n* = 30). In the group of 29 ^0/7^–32 ^6/7^ GA, the 5th percentile was defined as 1.0 g/L for fibrinogen, 24 IU/dL for FII and 41 IU/dL for factor V (*n* = 46). In the group of 33^0/7^–36^6/7^ GA, the 5th percentile was defined as 1.0 g/L for fibrinogen, 24 IU/dL for FII and 30 IU/dL for factor V (*n* = 46) (Table 2).

**Table 2:** Reference ranges for fibrinogen, factor II and factor V in preterm infants (*n* = 122)

	Fibrinogen (g/L) Median (5–95 percentile)	Factor II (IU/dL) Median (5–95 percentile)	Factor V (IU/dL) Median (5–95 percentile)
23 ^0/7^–28 ^6/7^ GA (*n* = 30)	1.5 (0.6–4.2)	33.0 (15–46)	62.5 (16–101)
29 ^0/7^–32 ^6/7^ GA (*n* = 46)	1.6 (1.0–3.6)	37.5 (24–52)	75.5 (41–120)
33 ^0/7^–36 ^6/7^ GA (*n* = 46)	1.8 (1.0–2.8)	38.0 (24–56)	78.0 (30–152)

Level of fibrinogen (g/L) was lower in case of IUGR (1.5 [1.2; 1.8] vs 1.8 [1.3; 2.2], *p* = 0.006). There was no difference regarding factors II and V.

### Correlations with biological data

Levels of fibrinogen (g/L), factor II (%) and factor V (%) were lower in case of thrombocytopenia (fibrinogen: 1.3 [1.0; 1.7] vs 1.8 [1.3; 2.2], p = < 0.001, factor II: 31.5 [25.0; 3.0] vs 38.0 [32.0; 46.0], *p* = 0.002 and factor V: 61.0 [43.0; 78.0] vs 74.5 [62.0; 90.0], *p* = 0.003).

Level of factor V (%) was lower in case of hypophosphatemia (61.5 [49.0; 79.0] vs 74.5 [64.0; 90.0], *p* = 0.015). There was no correlation between hypophosphatemia and fibrinogen and factor II (fibrinogen: *p* = 0.073, factor II: *p* = 0.43).

### Correlations with outcome

There was no association between birth platelets count and intraventricular hemorrhage development (*p* = 0.61). Factor II and fibrinogen at birth were also not associated with Intraventricular hemorrhage (Table 3). Only factor V level at birth was significantly associated with lower risk of lower in premature infants who developed intraventricular hemorrhage (63.5 [46.0; 76.5] vs 74.0 [58.0; 89.0], *p* = 0.026) (Table 3) with an unadjusted OR per SD increase in Factor V of 0.57 (95%CI, 0.34 to 0.96). After adjustment for age, the association between Factor V level and intraventricular hemorrhage was slightly attenuated (adjusted OR, 0.70; 95%CI, 0.40 to 1.23) but remained not significant (*p* = 0.22).There was no correlation with factor II and fibrinogen (Table 3).

**Table 3:** Platelets, fibrinogen, factor II and factor V according to IVH development

	IVH group (*n* = 28)	No IVH development (*n* = 94)	Unadjusted p
Platelets^a^ (G/L)	174.7 (72.5)	183.3 (79.2)	0.61
Fibrinogen (g/L)	1.8 [1.3; 2.1]	1.6 [1.2; 2.0]	0.63
Factor II (IU/dL)	32.0 [25.5; 44.0]	37.0 [31.0; 45.0]	0.21
Factor V (IU/dL)	63.5 [46.0; 76.5]	74.0 [58.0; 89.0]	0.026

Values are expressed as median [interquartile range] or mean (SD)

^a^: 3 missing values (3 newborns with no IVH development)

Correlations between coagulation factor’s levels and death could not be assessed given the small number of deaths.

## Discussion

Preterm birth is defined by the World Health Organization (WHO) as delivery before 37 completed weeks of gestation. Each year, 15 million babies are born preterm, which is estimated to be about 11% of all deliveries [13]. Among bleeding complications, IVH remains a significant complication of prematurity, with a large number of survivors sustaining neurodevelopmental sequelae [11]. The incidence of IVH reaches 45% in infants with birth weights less than 750 g, and 35% of these lesions are severe. IVH is typically diagnosed during the first days of life, 50% on the first day and 90% within the first 4 days [11]. The role of coagulation immaturity and platelet function in the pathogenesis of IVH is uncertain and still under debate. In addition, the definition of coagulopathy in premature newborns is not accurate. Standard coagulation assays, Prothrombin Time (PT) and Activated Partial Thromboplastin Time (APTT), don’t allow a reliable interpretation of hemostatic derangement, nor can provide a valid guidance for transfusion therapy. However, these neonates are sometimes transfused with fresh frozen plasma to prevent bleeding complications because of a perception that coagulation tests are abnormal. The specific dosages of FII, FV and fibrinogen may provide a more accurate evaluation of coagulopathies in the premature newborns but reference values for this very specific population are needed. Early in the 1980’s Andrews et al. established reference ranges in a large population of neonates born between 30 and 36 GA [5]. Due to recent progress in neonatal resuscitation, more premature and extremely premature infants can survive. Defining reference ranges of coagulation tests for infants < 30 GA is a challenge when severe bleedings are frequent.

In 1996, Reverdiau-Moalic et al. evaluated blood coagulation parameters in healthy human fetuses between 19 and 38 weeks’ gestation. The lower and upper boundaries including 95% of the population for fibrinogen (g/L) were as follows: 0.57–1.50 for fetuses of 19–23 GA (*n* = 20), 0.65–1.65 for fetuses of 24–29 GA (*n* = 22) and 1.25–1.65 for fetuses of 30–38 GA (n = 22). Regarding factor II and factor V, the lower and upper boundaries including 95% of the population were respectively as follows (%): 10–24 and 21–44 for fetuses of 19–23 GA (*n* = 20), 11–30 and 25–50 for fetuses of 24–29 GA (*n* = 22) and 15–50 and 23–70 for fetuses of 30–38 GA (*n* = 22). In each group, the lower boundary seems to be lower than in our study [7].

Further other studies examined coagulation factors in fetuses and concluded that birth process by itself, and especially the labor effect were essential in the maturation of the hemostatic system of the newborn [6].

Few studies have examined the levels of coagulation factors in premature infants shortly after birth. First reference ranges were published by Andrew in 1988 among 137 infants but were limited to healthy premature aged from 30 to 36 GA. The lower boundary was 1.5 g/L for fibrinogen, 20% for factor II and 41% for factor V [5]. Thanks to advances in neonatal resuscitation, more extremely premature infants can survive, and in the meantime, the reagents, automates and dosing techniques changed considerably. There is a need to update the standards and references in this population using appropriate reagents. In 2012, Poralla et al. examined the coagulation system of extremely preterm infants (23–27 GA). In very preterm infants, ranges of fibrinogen was between 0.93 and 1.36 g/L, of FII between 20 and 34% and of FV between 55 and 66% [14]. In 2014, Christensen described reference intervals for premature neonates with 5th and 95th percentiles. In the group < 28 weeks (*n* = 24), 5th percentile was 0.71 g/L for fibrinogen versus 0.87 g/L in the group 28–34 [15]. These data are consistent with those of our study.

We found in this study that levels of fibrinogen, factor II and factor V rose with increasing gestational age. This data seems consistent with the literature. Early in the 70’s Bleyer et al. described developmental hemostasis concept. They reported for the first time that the premature infant is born with low levels of nearly all the clotting factors known compared to the full term newborn [16]. It is nowadays well know that plasma levels of coagulation factors change as gestation progress related to fetal liver maturity.

We found that preterm neonates with IUGR had lower fibrinogen level at birth in consistence with previous studies [17, 18].

Franzoi et al. also reported that fibrinogen was more elevated in vaginally delivered neonates in comparison to those delivered by caesarean section but the difference was not statistically significant [19].

We found that factor V level at birth was correlated with presence of IVH. To the best of our knowledge, this is the first report of an association between factor V level at birth and IVH occurrence. However, in our study, the strength of association was slightly attenuated compared to unadjusted OR but remained not significant and a larger study is needed to establish if the factor V was an independent risk factor of IVH. Previous studies on the topic mainly focused on global coagulation tests. In 1982, Setzer reported that among premature the IVH group had a mean bleeding time which was significantly prolonged compared to that of the group without IVH [20]. Same, in 2015, Duppré et al. reported that INR levels were significantly increased in neonates with IVH of any grades [21]. Conversely, Christensen found that abnormal cord blood coagulation values at preterm birth did not predict bleeding during the first week [15].

The overall prevalence of thrombocytopenia in neonates admitted to NICUs ranges from 22 to 35%. In our study there was no significant association between thrombocytopenia and IVH. Conversely, in some studies thrombocytopenia was independently associated with IVH or IVH severity [22] whereas others found no relationship between the severity of thrombocytopenia and IVH [23, 24].

The place of fresh frozen plasma in the prevention of IVH is still unclear. The only randomized control trial assessing the effect of prophylactic fresh frozen plasma in 776 preterm babies reported no effect on early mortality and morbidity [25]. However this research was performed more than 20 years ago.

Some authors have even suggested that the expansion of blood volume could increase cerebral blood flow and cerebral capillary pressure, enough to rupture subependymal vessels [26]. Recent evidence-based reviews about neonatal plasma transfusion conclude that it seems to be no place for prophylactic plasma to prevent IVH [9].

Our study has limitations inherent to the method and strengths. Limitations included the retrospective design of the study and the lack of exhaustive coagulation screening. Another limitation of the study is that prematurity is an independent risk factor for IVH even in newborns with normal coagulation factor levels. The major strength of our study is the important effective of premature and the homogeneity of its population (without infection, without anoxia, without preeclampsia).

## Conclusion

In this study, we can define hemostatic profile among 122 newborns with spontaneous prematurity, corroborating references ranges for studied parameters. One of the important feature of this study was that factor V level at birth was correlated with IVH development.

Further large and prospective studies are still called for, to increase the number of patients and in particular in order to correlate the grade of hemorrhage and the factor V level at birth.

## Data Availability

All data generated or analysed during this study are included in this published article.
